# Comparative analysis of first-generation epidermal growth factor receptor inhibitors combined with chemotherapy versus third-generation epidermal growth factor receptor inhibitors in the treatment of advanced non-small cell lung cancer: a systematic review and meta-analysis

**DOI:** 10.3389/fphar.2025.1586332

**Published:** 2025-08-14

**Authors:** Siyan Peng, Zhixin Yu, Honglin Zhu, Chuwen Liang, Huijuan Qiu, Shaodong Hong, Yixin Zhou

**Affiliations:** ^1^ State Key Laboratory of Oncology in South China, Collaborative Innovation Center for Cancer Medicine, Sun Yat-sen University Cancer Center, Guangzhou, China; ^2^ Department of VIP Region, Sun Yat-sen University Cancer Center, Guangzhou, China; ^3^ Department of Medical Oncology, Sun Yat-sen University Cancer Center, Guangzhou, China

**Keywords:** EGFR mutant, chemotherapy, targeted therapy, advanced non small cell lung cancer, combined therapy

## Abstract

**Background:**

In advanced non-small cell lung cancer with EGFR mutations, third-generation EGFR TKIs (3^rd^-G TKIs) are currently the preferred first-line treatment. Previous studies have demonstrated that combining first-generation EGFR TKIs with chemotherapy (1^st^-G TKIs + chemo) also significantly enhances efficacy compared to 1^st^-G TKIs alone. This study aims to compare the effectiveness of 1^st^-G TKIs + chemo against 3^rd^-G TKIs.

**Methods:**

We conducted an indirect meta-analysis of randomized controlled trials comparing 1^st^-G TKIs + chemo to 3^rd^-G TKIs. Randomized controlled trials (RCTs) were searched from the PubMed, Embase and Cochrane Library databases. Outcomes included progression-free survival (PFS), overall survival (OS), objective response rate (ORR), and grade ≥3 treatment-related adverse events (TRAEs). Data were analyzed using inverse variance and Mantel-Haenszel methods.

**Results:**

Ten RCTs with 3,014 patients met the inclusion criteria. Direct comparisons indicated that 1^st^-G TKIs + chemo significantly improved PFS (HR 0.54, *P* < 0.001), OS (HR 0.62, *P* < 0.001), and ORR (RR 1.21, *P* < 0.001) compared to 1^st^-G TKIs alone. Indirect comparisons between 1^st^-G TKIs + chemo and 3^rd^-G TKIs revealed no significant differences in PFS (HR 1.17; 95% CI, 0.98 to 1.40; *P* = 0.075) or OS (HR 0.78; 95% CI, 0.56 to 1.07; *P* = 0.122). Although 1^st^-G TKIs + chemo showed a 16% improvement in ORR compared to 3^rd^-G TKIs (RR 1.16; 95% CI, 1.06 to 1.27; *P* < 0.001), it was also associated with a notable increase in grade ≥3 TRAEs (RR 2.41; 95% CI, 1.63 to 3.57; *P* < 0.001).

**Conclusion:**

1^st^-G TKIs + chemo demonstrated PFS and OS comparable to 3^rd^-G TKIs. Moreover, 1^st^-G TKIs + chemo may be a viable option for patients who prioritize a higher response rate.

**Systematic Review Registration:**

https://www.crd.york.ac.uk/PROSPERO/view/CRD42023461565 identifer, PROSPERO (CRD42023461565).

## 1 Introduction

Lung cancer is the predominant cause of cancer death worldwide, with approximately 85% of patients presenting with the histological subtypes of non-small cell lung cancer (NSCLC), the most common of which are lung adenocarcinoma (LUAD) and lung squamous cell carcinoma (LUSC) (Herbst et al., 2018). A substantial proportion of patients are unsuitable for surgical intervention, necessitating palliative internal medicine treatments. Epidermal growth factor receptor (EGFR) mutations occur in 40%–60% of Southeast Asian patients and 10%–20% of Caucasian patients with lung adenocarcinomas and reach occurrences of 50%–60% in non-smoking patients with lung cancer. The most common EGFR mutations include exon 19 deletions and missense mutations in exon 21 (Hsu et al., 2018).

Since 2009, the IPASS study (Mok et al., 2009) has validated the effectiveness of 1^st^-G TKIs for patients with EGFR-mutant NSCLC. Substantial advancements have been made in the first-line treatment of advanced EGFR-mutant NSCLC, particularly with the development of 3^rd^-G TKIs, as demonstrated by studies such as FLAURA (Soria et al., 2018), FLAURA China (Cheng et al., 2021), FURLONG (Shi et al., 2022), AENEAS (Lu et al., 2022) and LASER301 (Cho et al., 2023). These advancements have established 3^rd^-G TKIs as the standard of care. However, the resistance mechanisms to 3^rd^-G TKIs are complex, and subsequent treatment options remain limited (Fu et al., 2022). Additionally, the adoption of 3^rd^-G TKIs as first-line therapy has been constrained in certain regions due to lack of inclusion in insurance coverage, rendering it inaccessible for many patients. Moreover, the APPLE trial (Remon et al., 2024) indicated that sequential treatment with 1^st^-G TKIs followed by 3^rd^-G TKIs offers comparable overall survival (OS) benefits to upfront 3^rd^-G TKIs administration. This suggests a continued role for 1^st^-G TKIs and their combinations in clinical practice, highlighting the potential and necessity for combination therapies based on 1^st^-G TKIs.

Combination therapy with multiple drugs, particularly the addition of chemotherapy, is a pivotal approach to delaying the onset of drug resistance. Chemotherapy, as a systemic treatment, may eradicate subsets of cancer cells contributing to EGFR-TKIs resistance (Miller and Hanna, 2021). Several clinical studies have demonstrated that 1^st^-G TKIs + chemo significantly enhances efficacy compared to 1^st^-G TKIs alone. However, a direct comparison between 1^st^-G TKIs + chemo and 3^rd^-G TKIs has not yet been conducted.

To address this gap, we devised an indirect comparison using 1^st^-G TKIs as a bridge to compare the efficacy and safety of 1^st^-G TKIs + chemo *versus* 3^rd^-G TKIs in treating patients with advanced NSCLC harboring EGFR mutations. This study aims to provide additional comparative data to support informed decision-making in first-line therapy. Additionally, we performed subgroup analyses to identify patient populations that might derive greater benefit from 1^st^-G TKIs + chemo.

## 2 Methods

This meta-analysis followed the PRISMA (Page et al., 2021) guidelines and was registered on the PROSPERO website (CRD42023461565).

### 2.1 Data sources and searches

Relevant studies were comprehensively searched in PubMed, Embase, and Cochrane Library databases prior to 31 December 2023, using the following search terms: “epidermal growth factor receptor,” “non-small cell lung carcinoma,” “Gefitinib,” “Erlotinib,” “Icotinib,” “Chemotherapy,” “Osimertinib,” “Furmonertinib,” “Aumolertinib,” “Lazertinib,” “Olmutinib,” and “randomized clinical trial (RCT).” A detailed description of the retrieval method is provided in Supplementary Material 1. Two investigators (S.P and Z.Y) independently assessed the articles for eligibility, and disagreements between the investigators were resolved through further discussion with a third investigator.

### 2.2 Study selection

The inclusion criteria for study selection were as follows: 1) patients with advanced, EGFR-mutated NSCLC; 2) interventions: treatment with 1^st^-G TKIs + chemo *versus* a 1^st^-G TKIs as the control arm or treatment with a 3^rd^-G TKIs *versus* a 1^st^-G TKIs as the control arm; 3) Outcome Measures: inclusion of the hazard ratio (HR) and the corresponding 95% confidence interval (CI) for progression-free survival (PFS); and 4) randomized controlled trials.

### 2.3 Assessment of study quality

The risk of bias for each eligible study was evaluated using the Cochrane Risk of Bias Tool (Higgins et al., 2011).

### 2.4 Data extraction and outcomes

Two authors (S.P and Z.Y) independently extracted data and reached a consensus. The primary endpoint was PFS, while the secondary endpoints were OS, objective response rate (ORR), and grade ≥3 treatment-related adverse events (TRAEs). PFS and OS were collected as HRs with 95% CIs, while ORR and grade≥3 TRAEs were collected as the dichotomous data.

### 2.5 Data analyses

In the direct comparison of 1^st^-G TKIs + chemo or 3^rd^-G TKIs *versus* 1^st^-G TKIs, for continuous variables, which included PFS and OS, HRs with 95% CIs were assessed for each individual study. A meta-analysis of the HRs was performed using the inverse variance technique. Furthermore, for the analysis of ORRs, grade≥3 TRAEs, RRs, and corresponding 95% CIs were calculated for each study by adopting the Mantel-Haenszel method (DerSimonian and Laird, 1986). Heterogeneity was evaluated using Cochran’s Q test; a *P* value < 0.1 and *I* ^2^ > 50% indicated statistical heterogeneity, thus requiring the use of a random-effects model; otherwise, a fixed-effects model was used (Cumpston et al., 2019).

When conducting an indirect comparison between 1^st^-G TKIs + chemo and 3^rd^-G TKIs, an adjusted indirect comparison was performed on arm A (1^st^-G TKIs + chemo) *versus* arm B (3^rd^-G TKIs), linked by arm C (1^st^-G TKIs), using the frequentist methods with the following formula (Bucher et al., 1997): log *HR*_AB_ = log *HR*_AC_ ˗ log *HR*_BC_, and its standard error (SE) for the log *HR* was *SE* (log *HR*AB) = $\sqrt{{SE\left(\log\;HR\text{AC}\right)}^{2}+{SE\left(\log\;HR\text{BC}\right)}^{2}}$ . RR was evaluated similarly using this formula.

All statistical analyses were conducted using STATA software (version 17.0). Statistical significance was defined as a two-sided *P*-value < 0.05.

## 3 Results

### 3.1 Characteristics of the eligible studies

**FIGURE 1:**
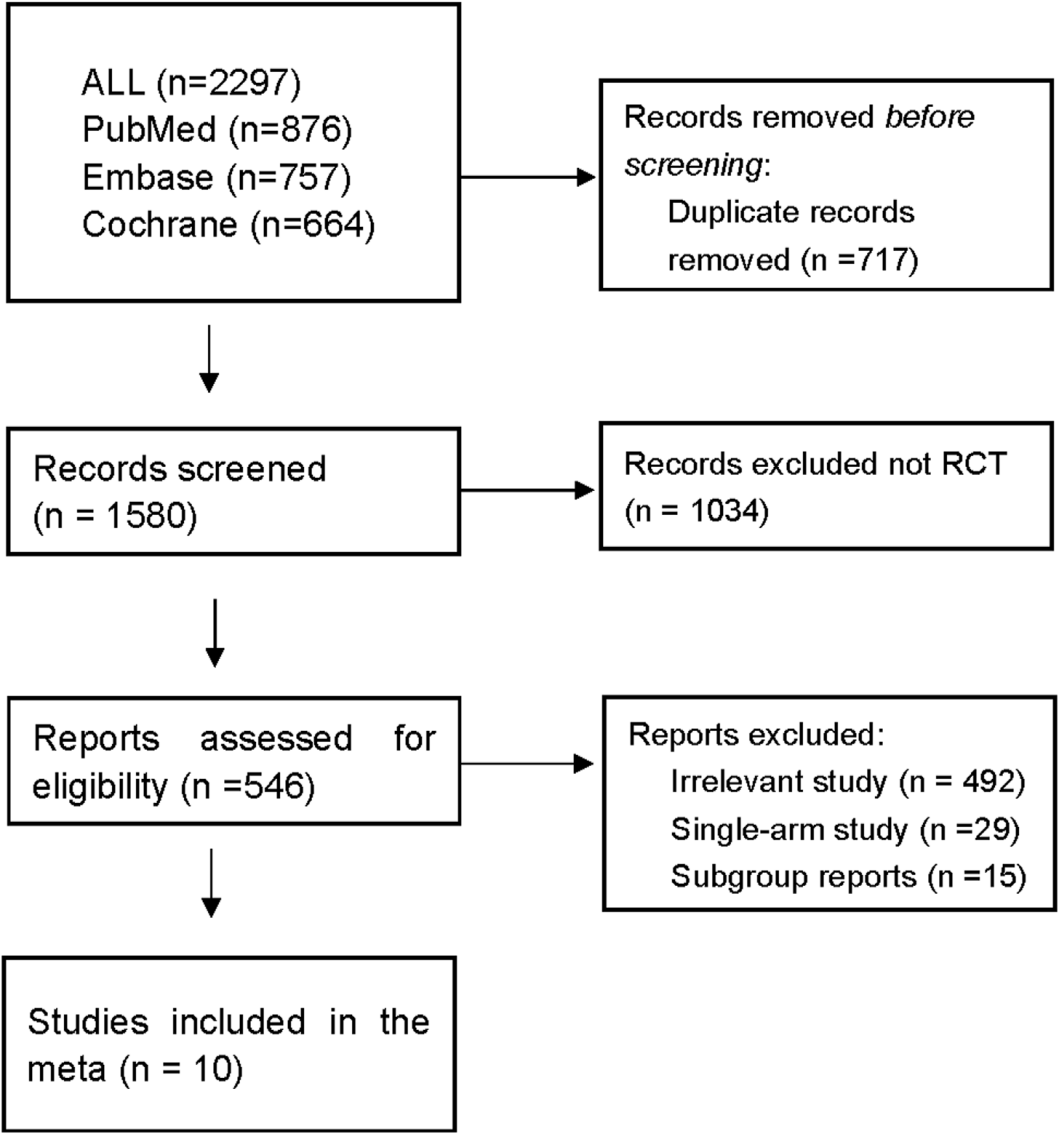
Flow diagram of trial selection.

Ten RCTs (Soria et al., 2018; Cheng et al., 2021; Shi et al., 2022; Lu et al., 2022; Cho et al., 2023; Xu et al., 2019; Cheng et al., 2016; Noronha et al., 2020; Hosomi et al., 2020; Han et al., 2017) were deemed eligible for inclusion in the meta-analysis and included a total of 3014 patients. A brief summary of the included studies is presented in Table 1, and the search process is described in Figure 1. Of the included studies, five investigated the efficacy of 1^st^-G TKIs + chemo (n = 600) *versus* 1^st^-G TKIs (n = 543), while the remaining five trials (Soria et al., 2018; Cheng et al., 2021; Shi et al., 2022; Lu et al., 2022; Cho et al., 2023) explored 3^rd^-G TKIs (n = 938) *versus* 1^st^-G TKIs (n = 933). There were two randomized phase II Trials (Cheng et al., 2016; Han et al., 2017) and eight randomized phase III studies. The primary endpoint of all studies was PFS, and the PFS of 1^st^-G TKIs fluctuated between 8 and 11.9 months. The PFS durations observed for 1^st^-G TKIs + chemo spanned from 15.8 to 20.9 months, while the PFS profiles for 3^rd^-G TKIs ranged from 17.8 to 20.8 months.

**TABLE 1:** Characteristics of the included randomized controlled trials.

Study	Year	Phase	Treatment strategies	Sample size	Primary endpoint	PFS (months)	OS (months)	ORR(%)	PFS HR (95% CI)	OS HR (95% CI)
NCT02031601	2019	III	Icotinib + CT	90	PFS	16.0	36.0	78	0.59(0.42–0.84)	0.81(0.54–1.22)
			Icotinib	89		10.0	34.0	64		
NCT01469000	2016	II	Gefinitib + CT	126	PFS	15.8	43.4	80	0.68(0.48–0.96)	0.77(0.50–1.20)
			Gefinitib	65		10.9	36.8	74		
CTRI/2016/08/007,149	2019	III	Gefinitib + CT	174	PFS	16.0	NR	75	0.51(0.39–0.66)	0.45(0.31–0.65)
			Gefinitib	176		8.0	17.0	63		
NEJ009	2019	III	Gefinitib + CT	170	PFS,OS	20.9	50.9	84	0.49(0.39–0.62)	0.72(0.55–0.95)
			Gefinitib	172		11.9	38.8	67		
NCT02148380	2017	II	Gefinitib + CT	40	PFS	17.5	32.6	83	0.48(0.29–0.78)	0.36(0.20–0.67)
			Gefinitib	41		11.9	25.8	66		
FLAURA	2018	III	Osimertinib	279	PFS	18.9	38.6	80	0.46(0.37–0.57)	0.80(0.64–1.00)
			1st-G TKI	277		10.2	31.8	76		
FLAURA China	2021	III	Osimertinib	71	PFS	17.8	33.1	NR	0.56(0.37–0.85)	0.85(0.56–1.29)
			1st-G TKI	65		9.8	25.7	NR		
FURLONG	2022	III	Furmonertinib	178	PFS	20.8	NR	89	0.44(0.34–0.58)	NR
			Gefinitib	179		11.1	NR	84		
AENEAS	2022	III	Aumolertinib	214	PFS	19.3	NR	74	0.46(0.36–0.60)	NR
			Gefinitib	215		9.9	NR	72		
LASER301	2022	III	Lazertinib	196	PFS	20.6	26.7-NR	76	0.45(0.34–0.58)	0.74(0.51–1.08)
			Gefinitib	197		9.7	23.8-NR	76		

Abbreviations: PFS, progression-free survival; OS, overall survival; ORR, objective response rate; HR, hazard ratio; CT, chemotherapy; 1^st^ -G TKI, first-generation epidermal growth factor receptor tyrosine kinase inhibitor; NR, not reported.

A summary of the risk of bias evaluation is provided in Supplementary Material 2. The primary source of bias stemmed from the absence of blinding in clinical studies that compared 1^st^-G TKIs + chemo with 1^st^-G TKIs. Funnel plots are shown in Supplementary Material 3, with no asymmetry observed in the funnel plots. (PFS Egger test *P* = 0.107, OS Egger test *P* = 0.237, ORR Egger test *P* = 0.105, grade ≥3 TRAEs Egger test *P* = 0.164).

### 3.2 Direct comparisons between 1st-G TKIs + chemo or 3rd-G TKIs *versus* 1st-G TKIs

**FIGURE 2:**
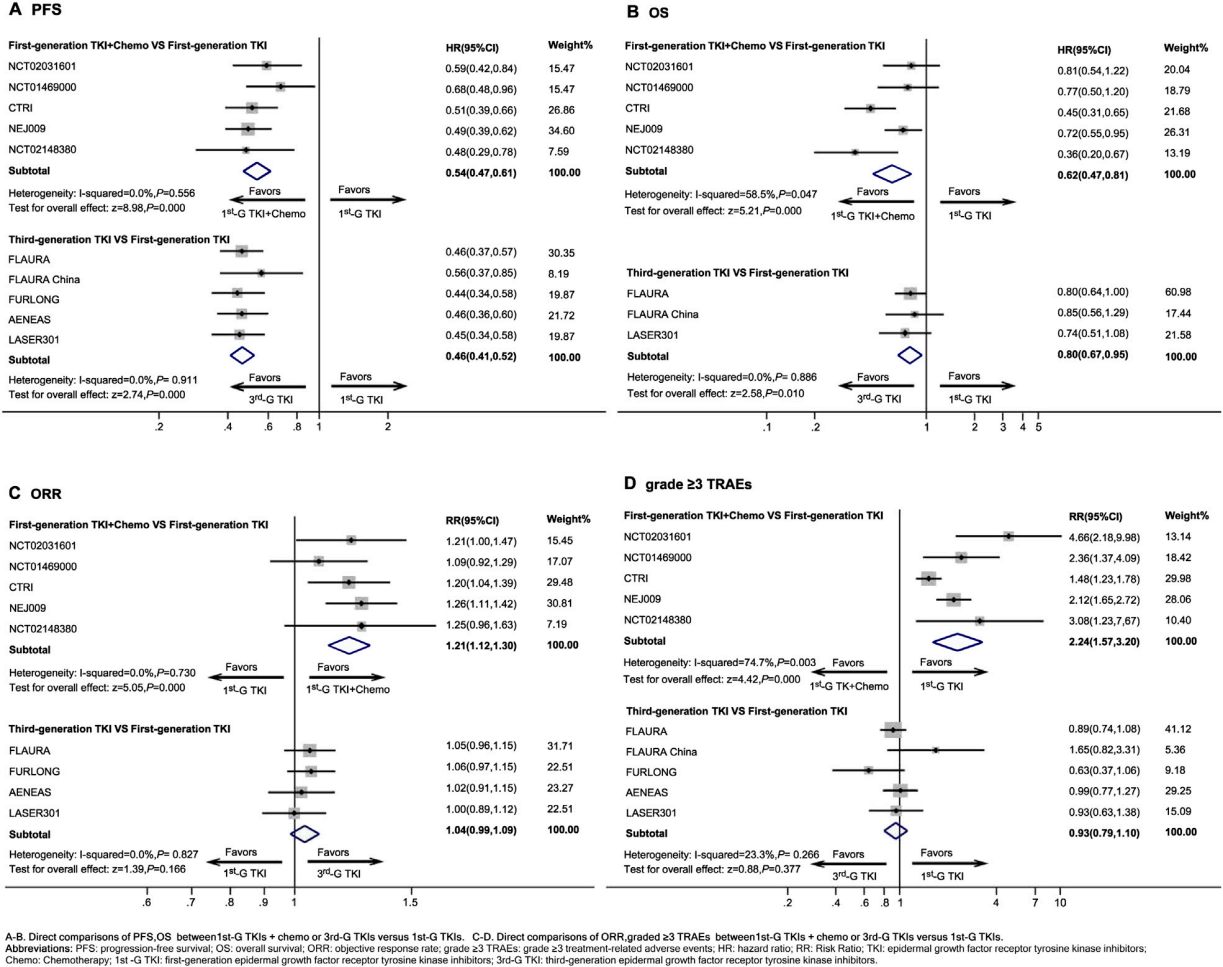
Direct comparisons between first-generation EGFR-TKIs plus chemotherapy or third-generation EGFR-TKIs *versus* first-generation EGFR-TKIs Abbreviations: PFS, progression-free survival; OS, overall survival; ORR, objective response rate; grade ≥3 TRAEs, grade ≥3 treatment-related adverse events; HR, hazard ratio; RR: Risk Ratio; TKI, epidermal growth factor receptor tyrosine kinase inhibitors; Chemo, Chemotherapy; 1^st^-G TKI, first-generation epidermal growth factor receptor tyrosine kinase inhibitors; 3^rd^-G TKI, third-generation epidermal growth factor receptor tyrosine kinase inhibitors.

1^st^-G TKIs + chemo presented a significant improvement in PFS (HR, 0.54; 95%CI, 0.47 to 0.61, *P* < 0.001) (Figure 2A), OS (HR, 0.62; 95%CI, 0.47 to 0.81, *P* < 0.001) (Figure 2B), and ORR (RR, 1.21; 95%CI, 1.12 to 1.30, *P* < 0.001) (Figure 2C) compared to use of 1^st^-G TKIs as monotherapy. However, the risk of grade ≥3 TRAEs (RR, 2.24; 95%CI, 1.57 to 3.20, *P* < 0.001) (Figure 2D) of 1^st^-G TKIs + chemo was increased compared to 1^st^-G TKIs monotherapy.

Compared with 1^st^-G TKIs, 3^rd^-G TKIs significantly improved PFS (HR, 0.46; 95%CI, 0.42 to 0.52, *P* < 0.001) (Figure 2A) and showed a significant improvement in OS (HR, 0.80; 95%CI, 0.67 to 0.95, *P* = 0.010) (Figure 2B), although there was no statistically significant difference in ORR (RR, 1.04; 95%CI, 0.99 to 1.09, *P* = 0.166) (Figure 2C). The risk of grade ≥3 TRAEs (RR, 0.93; 95%CI, 0.79 to 1.10, *P* = 0.377) (Figure 2D) was similar between 1^st^-G TKIs and 3^rd^-G TKIs regimens.

### 3.3 Indirect comparisons between 1^st^-G TKIs + chemo and 3^rd^-G TKIs

**FIGURE 3:**
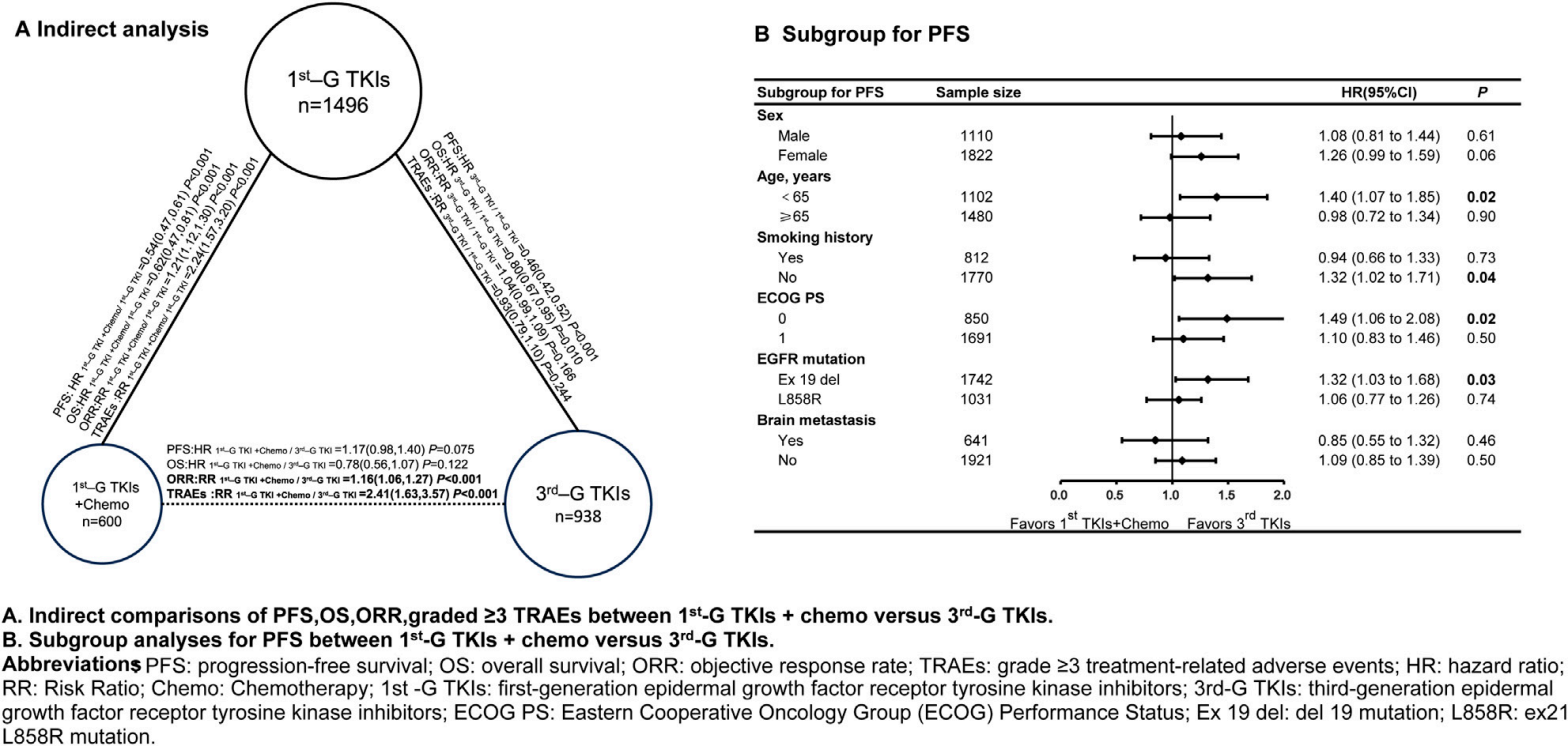
Indirect comparisons and subgroup analyses for PFS between first-generation EGFR-TKIs plus chemotherapy and third-generation EGFR-TKIs Abbreviations: PFS, progression-free survival; OS, overall survival; ORR, objective response rate; TRAEs, grade ≥3 treatment-related adverse events; HR, hazard ratio; RR, Risk Ratio; Chemo, Chemotherapy; 1^st^ -G TKI, first-generation epidermal growth factor receptor tyrosine kinase inhibitors; 3^rd^-G TKI, third-generation epidermal growth factor receptor tyrosine kinase inhibitors; ECOG PS, Eastern Cooperative Oncology Group (ECOG) Performance Status; Ex 19 del, del 19 mutation; L858R, ex21 L858R mutation.

1^st^-G TKIs + chemo presented numerically poorer PFS than 3^rd^-G TKIs regimens (HR, 1.17; 95%CI, 0.98 to 1.40, *P* = 0.075) (Figure 3A), although this difference lacks statistical significance. Across most subgroups, a shorter PFS was observed in 1^st^-G TKIs + chemo compared to the 3^rd^-G TKIs, particularly in patients younger than 65 years of age (HR, 1.40; 95%CI, 1.07 to 1.85, *P* = 0.02), patients with no history of smoking (HR, 1.32; 95%CI, 1.02 to 1.71, *P* = 0.04), patients with a PS score of 0 (HR, 1.49; 95%CI, 1.06 to 2.08, *P* = 0.02), and patients with EGFR exon 19 deletion mutations (HR, 1.32; 95%CI, 1.03 to 1.68, *P* = 0.03) (Figure 3B). However, in patients with brain metastases (HR, 0.85; 95%CI, 0.55 to 1.32, *P* = 0.46) (Figure 3B), the efficacy of 1^st^-G TKIs + chemo was numerically longer than that of 3^rd^-G TKIs, without statistical significance.

Conversely, 1^st^-G TKIs combined with chemotherapy produced a numerically lower risk of death than 3^rd^-G TKIs (OS: HR 0.78, 95% CI 0.56–1.07; *P* = 0.122), but this difference did not reach statistical significance (Figure 3A). The combination did, however, yield a significantly higher objective response rate (ORR: RR 1.16, 95% CI 1.06–1.27; *P* < 0.001) and was associated with a markedly increased incidence of grade ≥3 treatment-related adverse events (RR 2.41, 95% CI 1.63–3.57; *P* < 0.001) (Figure 3A).

## 4 Discussion

To the best of our knowledge, this is the first meta-analysis to perform an indirect comparison between 1^st^-G TKIs + chemo and 3^rd^-G TKIs alone. Our findings indicate that PFS and OS were comparable between these two regimens in patients with EGFR-mutant NSCLC. However, the 1^st^-G TKIs + chemo significantly increased the ORR by 16%, at the cost of a marked increase in the risk of grade ≥3 TRAEs (RR, 2.41, *P* < 0.001).

From the meta-analysis of the direct comparison between 1^st^-G TKIs + chemo and the 1^st^-G TKIs, 1^st^-G TKIs + chemo presented significantly longer PFS and OS than 1^st^-G TKIs alone, in agreement with the findings of previous meta-analyses (Wu et al., 2021). A possible reason for this is that some patients treated with 1^st^-G TKIs alone did not receive subsequent chemotherapy after disease progression, for various reasons. Moreover, early combination chemotherapy can delay resistance to EGFR-TKIs and improve the survival rate of patients compared to sequential chemotherapy (Chang et al., 2021). The possible mechanism is that tumors with acquired drug resistance may contain a mixed cell population of drug-sensitive and drug-resistant cells with different growth rates, and early combined chemotherapy can better eliminate this heterogeneity (Moore and Wheatley-Price, 2021).

Furthermore, in the indirect comparison, when the 1^st^-G TKIs was replaced with the current preferred 3^rd^-G TKIs, the PFS and OS benefits of 1^st^-G TKIs + chemo compared to 3^rd^-G TKIs were comparable, indicating that1^st^-G TKIs + chemo has potential as a first-line treatment. However, it is important to note that in studies of 1^st^-G TKIs + chemo, only 23.3% of patients subsequently received 3^rd^-G TKIs treatment (Hosomi et al., 2020). If more patients were able to receive 3^rd^-G TKIs after progression, the results of 1^st^-G TKIs + chemo would likely be even more pronounced.

Despite these potential benefits, 1^st^-G TKIs + chemo has several notable drawbacks. These include increased adverse reactions and the possibility of rapid disease progression or death in some patients, preventing them from accessing 3^rd^-G TKIs. Therefore, careful consideration and patient selection are crucial. Identifying subgroups of patients who are more likely to benefit from 1^st^-G TKIs + chemo is essential to optimize treatment outcomes.

From the results of the primary endpoint, 1^st^-G TKIs + chemo presented a statistically significant advantage in terms of ORR compared with 3^rd^-G TKIs. This indicates that the use of 1^st^-G TKIs + chemo can effectively reduce the tumor burden and is suitable for patients requiring rapid tumor shrinkage. Based on the subgroup analysis, it is noteworthy that, among patients with baseline brain metastases, first-generation EGFR-TKI plus chemotherapy yielded numerically longer progression-free survival (HR 0.85, 95% CI 0.55–1.32; P = 0.46); however, this difference was not statistically significant, and no superiority over third-generation EGFR-TKIs can be claimed. Osimertinib and other third-generation TKIs are known to reach higher cerebrospinal-fluid concentrations than first-generation compounds (Popat et al., 2023), and any apparent advantage of adding chemotherapy remains speculative and requires dedicated pharmacokinetic investigation. Furthermore, our meta-analysis lacked individual-patient data on intracranial progression; future studies should report CNS-specific endpoints such as CNS progression-free survival or time-to-brain-progression to determine the relative efficacy of these strategies in patients with brain metastases.

In other subgroups, including patients younger than 65 years, non-smokers, those with an ECOG PS of 0, and those with EGFR exon 19 deletion mutations, 3^rd^-G TKIs were the preferred treatment. EGFR mutation type is a crucial biomarker for treating NSCLC with EGFR-TKIs. Compared to 1^st^-G TKIs + chemo, patients with EGFR exon 19 deletion achieved a superior PFS (HR, 1.32; 95% CI, 1.03–1.68) when treated with 3^rd^-G TKIs as monotherapy, while patients with exon 21 mutation showed comparable PFS (HR, 1.06; 95% CI, 0.77–1.39) when treated with 1^st^-G TKIs + chemo and 3^rd^-G TKIs. This finding could be attributed to the higher incidence of the T790M mutation and the lower incidence of concomitant mutations in patients with EGFR exon 19 deletions than in those with EGFR exon 21 mutations, therefore, the selection of a 3^rd^-G TKIs could directly overcome the T790M mutation, thereby extending PFS (Hong et al., 2018). Moreover, In patients aged <65 years, never-smokers, and ECOG 0, several biological and clinical factors may underlie the observed superiority of third-generation EGFR-TKIs. First, never-smokers harbour markedly fewer tobacco-related passenger mutations and a lower prevalence of resistance-associated co-mutations such as TP53, STK11 and RB1, which are enriched in smoking-related tumours and accelerate escape from EGFR blockade (Moorthi et al., 2024; Labbe et al., 2017). Second, the absence of heavy mutagenic exposure yields a comparatively low tumour-mutational burden, reinforcing ‘oncogene addiction’ to mutant EGFR and prolonging sensitivity to potent third-generation inhibitors (Rudin et al., 2009). Third, patients with ECOG 0 generally tolerate therapy without dose reduction, preserving full osimertinib exposure; dose-intensity analyses from AURA3 and real-world series confirm that optimal pharmacokinetics translate into deeper and more durable responses (Papadimitrakopoulou et al., 2020; Ramalingam et al., 2020). Although compelling, these subgroup findings remain exploratory and should be validated prospectively, ideally in trials stratified by co-mutation status and performance score.

The recent study FLAURA2 (Planchard et al., 2023) that compared osimertinib combined with chemotherapy to 1^st^-G TKIs monotherapy revealed that the osimertinib regimen presented a significant improvement in progression-free survival (PFS) (25.5 months vs. 16.7 months) among patients with advanced NSCLC and EGFR mutations. This suggests that combining 3^rd^-G TKIs and chemotherapy may offer a new treatment option for these patients. However, subsequent OS data must be considered to determine this regimen’s clinical significance. The combination of EGFR-TKI with other drugs will be a focus of future research and may impact the current standard of using 3^rd^-G TKIs as a first-line therapy.

Based on the results of our current study, considering both efficacy and safety data, 1^st^-G TKIs + chemo did not present superior performance over 3^rd^-G TKIs. However, 1^st^-G TKIs + chemo is a potential choice for patients with brain metastases or those who require high response rates.

Our study has some limitations that should be noted when interpreting our findings. First, the number of included studies was relatively small, and only two phase II studies were present in our dataset, which reduced the level of evidence. Second, no direct comparative study was available. In addition, OS and ORR were not the primary endpoints; thus, the data may be considered immature. Finally, we were unable to further compare 1^st^-G TKIs + chemo and 3^rd^-G TKIs + chemo groups.

In this meta-analysis, 1^st^-G TKIs + chemo regimens presented PFS and OS comparable to those of 3^rd^-G TKIs, with a statistically significant improvement in ORR. However, the risk of grade ≥3 TRAEs significantly increased. This suggests that in clinical practice, for patients with EGFR mutations, if we intend to use 1^st^-G TKIs + chemo as a first-line treatment, further population screening is imperative to determine the optimal medication.

## Data Availability

The original contributions presented in the study are included in the article/Supplementary Material, further inquiries can be directed to the corresponding author.
