# Supplementary material for: Comparative analysis of first-generation epidermal growth factor receptor inhibitors combined with chemotherapy versus third-generation epidermal growth factor receptor inhibitors in the treatment of advanced non-small cell lung cancer: a systematic review and meta-analysis
Source: Front Pharmacol. 2025 Aug 14;16:1586332. doi: 10.3389/fphar.2025.1586332 (PMC12391186; doi:10.3389/fphar.2025.1586332)
Supplement: Supplementary file 1 [file DataSheet1.pdf]

Supplementary file 1

retrieval methods for PubMed, Embase, and Cochrane database

PubMed (876)

("EGFR"[Title/Abstract] OR "epidermal growth factor receptor"[Title/Abstract] OR "EGFR-Mutated"[Title/Abstract] OR "mutated epidermal growth factor receptor" [Title/Abstract] OR "egfr mutations"[Title/Abstract]) AND （"NSCLC"[Title/Abstract] OR "non small cell lung carcinoma"[Title/Abstract] OR "carcinoma, non small cell lung"[MeSH Terms]） AND ((("Gefitinib"[Title/Abstract] OR "Erlotinib"[Title/Abstract] OR "Icotinib"[Title/Abstract]) AND "Chemotherapy"[Title/Abstract]) OR ("Osimertinib" [Title/Abstract] OR "Furmonertinib"[Title/Abstract] OR "Aumolertinib"[Title/Abstract] OR "Lazertinib"[Title/Abstract] OR "Olmutinib"[Title/Abstract])) AND ("randomized clinical trial"[Title/Abstract] OR "phase"[Title/Abstract] OR "clinical trials as topic"[MeSH Terms])

Embase (757)

('egfr'/exp OR 'egfr' OR 'epidermal growth factor receptor':ab,ti OR 'egfr-mutated':ab,ti) AND ('nsclc' OR 'non-small cell lung cancer':ab,ti OR 'non small cell lung carcinoma':ab,ti) AND (('gefitinib':ab,ti OR 'erlotinib':ab,ti OR 'icotinib':ab,ti) AND 'chemotherapy':ab,ti OR 'osimertin ib':ab,ti OR 'furmonertinib':ab,ti OR 'aumolertinib':ab,ti OR 'lazertinib':ab,ti OR 'olmutinib':ab,t i) AND ('randomized controlled trial'/exp OR 'randomized controlled trial')

Cochrane (664 Trial)

#1 NSCLC OR non-small cell lung carcinoma OR non-small cell lung carcer OR non-squamous cell lung carcinoma

#2 EGFR-Mutated OR mutated epidermal growth factor receptor OR egfr mutations

#3 gefitinib OR erlotinib OR icotinib

#4 Chemotherapy OR pemetrexed OR carboplatin

#5 osimertinib OR furmonertinib OR aumolertinib OR lazertinib OR olmutinib

#6 #1 AND #2 AND #3 AND #4

#7 #1 AND #2 AND #5

Supplementary file 2 Summary of risk-of-bias analysis.

+ Low
− Unclear
X High

	Random sequence generation	Allocation concealment	Blinding of participants	Blinding of outcome assessment	Incomplete outcome data	Selective reporting	Other bias	Overall
NCT02031601	+	+	X	−	+	+	+	X
NCT01469000	+	−	X	−	+	+	+	X
CTRI/2016/08/007149	+	−	X	−	+	+	+	X
NEJ009	+	−	X	−	+	+	+	X
NCT02148380	+	−	X	−	+	+	+	X
FLAURA	+	+	+	+	+	+	+	+
FLAURA China	+	+	+	+	+	+	+	+
FURLONG	+	+	+	+	+	+	+	+
AENEAS	+	+	+	+	+	+	+	+
LASER301	+	+	+	+	+	+	+	+

Supplementary file 3 Funnel plots

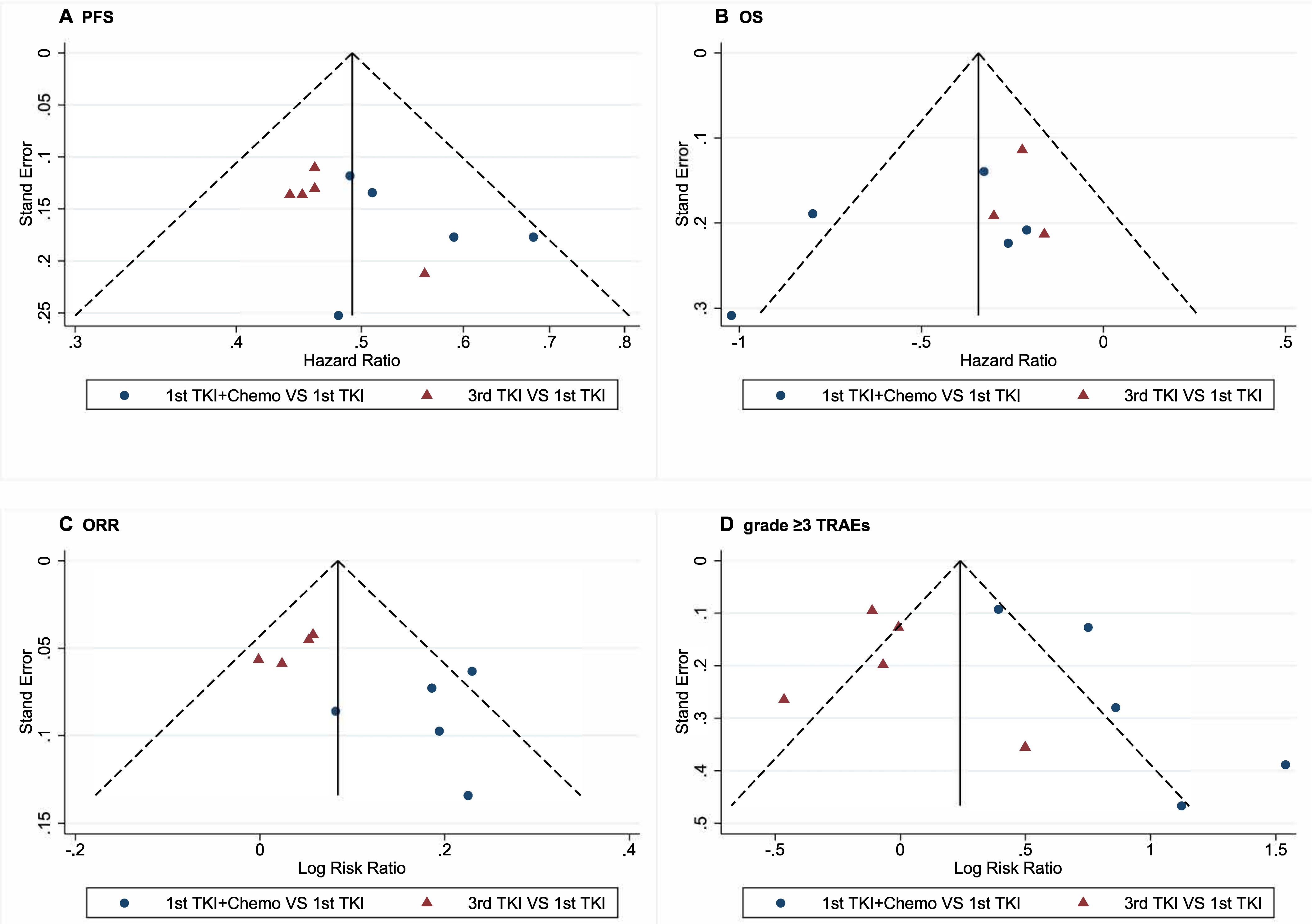

Abbreviations: PFS: progression-free survival; OS: overall survival; ORR: objective response rate; grade ≥3 TRAEs: grade ≥3 treatment-related adverse events; Chemo: Chemotherapy; 1st TKI: first-generation epidermal growth factor receptor tyrosine kinase inhibitors; 3rd TKI: third-generation epidermal growth factor receptor tyrosine kinase inhibitors.
